# Utilization of targeted sequencing for etiological diagnosis of pulmonary infections in different samples

**DOI:** 10.3389/fcimb.2025.1683489

**Published:** 2025-10-22

**Authors:** Xiaojun Guan, Kaisar Gufur, Liangliang Xu, Cuncun Chen, Ning Yu, Yi Fu, Mingjie Zhou, Abla Nurmamat

**Affiliations:** Department of Thoracic Surgery, Xinjiang Uygur Autonomous Region Sixth People’s Hospital, Urumqi, Xinjiang Uygur Autonomous Region, China

**Keywords:** targeted next-generation sequencing, pulmonary infection, pathogen detection, traditional pathogen detection methods, diagnostic value

## Abstract

**Objective:**

This study aims to assess the diagnostic value of targeted next-generation sequencing (tNGS) for pathogen identification from multiple sample types in patients with pulmonary infection, and to provide an alternative diagnostic method for clinical practice.

**Methods:**

Clinical data were collected from patients with suspected of pulmonary infection at the Thoracic Surgery Center of the Xinjiang Uygur Autonomous Region Sixth People’s Hospital. Samples, including bronchial lavage fluid (BALF), fresh tissue, pleural effusion, and sputum, were collected by attending physicians based on the patients’ clinical conditions. A total of 166 patients were enrolled, and their samples were subjected to pathogen detection using both tNGS and traditional pathogen detection methods (TPDs). The pathogen detection performance of tNGS was then compared with that of TPDs.

**Result:**

The positive detection rate of tNGS was significantly higher than that of TPDs (81.33% vs. 32.53%, p < 0.001). Among the 166 samples, tNGS identified a total of 65 pathogens, whereas TPDs identified only 14 (11 bacterial species, 2 fungal species, and *Mycoplasma pneumoniae*). TPDs primarily identified bacteria (including *Mycobacterium tuberculosis*) and fungi, and were unable to detect viruses. In contrast, tNGS revealed a broader spectrum of pathogens, including 35 bacterial species, 10 fungal species, 18 viral species, as well as *Mycoplasma pneumoniae* and *Chlamydia pneumoniae*. Notably, tNGS demonstrated greater efficiency in detecting mixed infections and further identified 16 antibiotic resistance genes (ARGs).

**Conclusion:**

tNGS exhibits higher sensitivity, a broader pathogen detection spectrum, and enhanced capability to identify mixed infections, along with the ability to detect ARGs. These advantages establish tNGS as a promising and reliable diagnostic modality for patients with pulmonary infections.

## 1 Introduction

Pulmonary infection is an inflammatory condition caused by pathogens such as bacteria, fungi, and viruses that invade the lung parenchyma or interstitium. This condition exhibits a high incidence and ranks among the primary contributors to clinical mortality worldwide (Febbo and Dako, 2024). Research indicates that nearly half of patients with pulmonary infections lack definitive identification of the causative pathogen (Gu et al., 2021), which underscores the urgency of developing sensitive, rapid, and highly specific detection modalities accurate diagnosis and optimal clinical management. Traditional pathogen detection methods (TPDs) are inherently limited by prolonged detection turnaround times and low positivity rates. These challenges frequently compel the adoption of empirical antibiotic therapy in clinical practice, which not only elevates the risk of the emergence of drug-resistant bacteria but may also exert adverse effects on patient outcomes (Chen et al., 2022). Currently, widely employed diagnostic methods for pathogens detection in pulmonary infections include smear microscopy, culture techniques, and immunological assays (Almas et al., 2023). However, each of these modalities suffers from distinct drawbacks, such as suboptimal sensitivity or limited specificity. Therefore, the implementation of timely and effective pathogen detection methods is crucial for enhancing diagnostic accuracy, guiding targeted therapy, and reducing the misuse of antibiotics.

Targeted next-generation sequencing (tNGS) represents an innovative high-throughput sequencing technology capable of both pathogens identification and characterization of antibiotic resistance genes (ARGs). This method integrates ultra-multiplex polymerase chain reaction (PCR) with high-throughput sequencing technology, thereby enabling the rapid and precise identification of a broad spectrum of known pathogenic microorganisms and their associated ARGs. Consequently, tNGS plays a pivotal role in the etiological diagnosis of respiratory tract infections. Currently, most research utilizing tNGS for pulmonary infection pathogen detection have focused on bronchoalveolar lavage fluid (BALF) specimens. However, additional high-quality clinical evidence is urgently needed to validate its diagnostic efficacy in this domain. To address this gap, we collected fresh tissue specimens, pleural effusion, BALF and sputum samples from patients with suspected pulmonary infections. We then performed tNGS in parallel TPDs to assess the clinical utility of tNGS in these patient population, with the aim of establishing an evidence base for guiding rational antimicrobial therapy.

## 2 Materials and methods

### 2.1 Patients and study design

**Figure 1:**
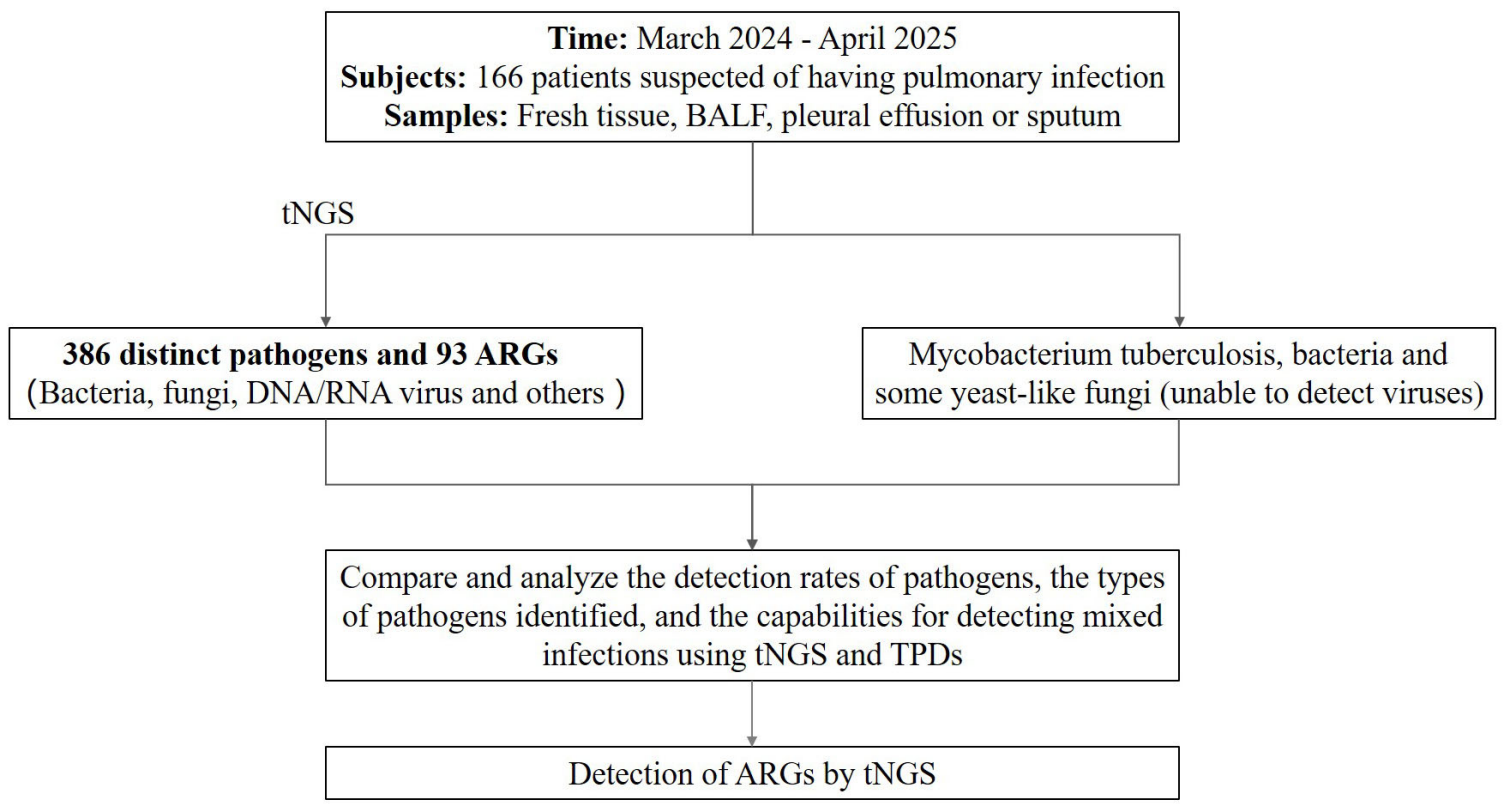
The layout of this study. BALF, bronchial lavage fluid; tNGS, targeted next-generation sequencing; TPDs, traditional pathogen detection methods; ARGs, antibiotic resistance genes.

A prospective study was conducted from March 2024 to April 2025 to evaluate the diagnostic efficacy of tNGS in patients suspected pulmonary infections. A total of 166 patients presenting with clinical signs and symptoms indicative of pulmonary infections were enrolled in this study. Each participant underwent both tNGS and TPDs for parallel diagnostic comparison. This study was approval by the Ethics Committee of the Xinjiang Uygur Autonomous Region Sixth People’s Hospital (Approval ID: 2025-051). The layout of this study is depicted in **Figure 1**.

### 2.2 Sample collection

Following obtaining written informed consent from all enrolled patients, attending physicians collected specimens (including BALF, fresh tissue, pleural fluid, or sputum) in accordance with patient-specific clinical protocols. Each sample was aliquoted into two portions, which were immediately transferred to sterile containers. One portion was sent to the Kindstar Global Precision Medicine Institute (Wuhan, China) for tNGS testing, while the other was subjected to TPDs analysis. All samples were stored at -80°C for future use.

### 2.3 Traditional pathogen detection methods

Collected samples were immediately placed in sterile, sealed containers to prevent microbial contamination and preserve activity and then transported to the hospital laboratory via a dedicated biological transport box. For *Mycobacterium tuberculosis* detection, acid-fast staining is first used for microscopic screening of red acid-fast bacilli. Meanwhile, samples are inoculated onto Löwenstein-Jensen medium and incubated at 37 °C (5%–10% CO_2_) for 4 to 8 weeks, during which colony morphology is observed for further identification. For other bacteria and fungi, pure cultures are prepared into standard suspensions and introduced to dedicated identification cards for VITEK-2 (bioMérieux). This instrument detects their reactions to biochemical substrates and compares the results with its built-in microbial database for precise identification. Positive controls (*E. coli* ATCC 25922) and negative controls (sterile saline) are set throughout the process to ensure test result reliability. If the test results show the presence of two or more pathogens, it is classified as a mixed infection.

### 2.4 tNGS data analysis

The pathogen detection panel enables the identification of 386 distinct pathogens and 93 ARGs (see **Supplementary Table S1** for the detailed list of detectable pathogens and resistance genes), including 85 Gram-positive bacteria, 74 Gram-negative bacteria, 71 fungi, 25 DNA viruses, 66 RNA viruses, 53 other pathogens (e.g., parasites and mycoplasmas), and 12 genera.

### 2.5 Library construction and sequencing

For sample pretreatment: BALF was centrifuged at 3000g for 10 min to harvest the pellet, which was then resuspended for concentration. Fresh tissues were minced into small pieces, homogenized using a tissue homogenizer, and centrifuged to recover the pellet. Pleural effusion was centrifuged at 3000×g for 15 min; the resulting pellet was resuspended and subjected to a second centrifugation for pathogen enrichment. Sputum samples were lysed with 0.1% dithiothreitol (DTT), centrifuged, and the pellet was washed to remove impurities. Following pretreatment, total DNA and RNA were extracted using the RNA/DNA Isolation Kit (R0017M, Beyotime, China). Library construction was performed with the 300+ Pathogen-Targeted Gene Detection Kit (Pathogeno, China); its core component, the P0–1806 Panel Mix, comprises a specific primer set capable of detecting over 300 pathogen species and ARGs. Two rounds of PCR were conducted to generate libraries: In the first round, multiplex PCR was carried out using specific primers that target conserved regions of pathogens and ARGs. Post-amplification, the PCR products were purified with magnetic beads. The second round of PCR involved amplification with primers harboring sequencing adapters and unique barcodes to enable sample differentiation. The amplified products were further purified through 1% agarose gel electrophoresis, and the quality of the library was assessed using a Qubit 4.0 Fluorometer (Thermo Scientific, USA). Typically, the library fragment size was around 350 bp, with a concentration of ≥ 1.0 ng/μL. Finally, sequencing was performed on the Illumina MiniSeq platform using the Universal Sequencing Kit (KS107-CXR, KingCreate, China).

### 2.6 Quality control and bioinformatic analyses

Internal controls and negative controls were included in each batch of samples for stringent quality control, with each sample generating >100,000 raw reads and a Q30 score of >85%. Adapter sequences were trimmed using Trimmomatic (v0.39), followed by filtering of reads <60 bp and low-quality sequences (Phred score <20); filtered reads were assigned to specific microbial taxonomic units via Kraken2 (v2.1.2, incorporating the NCBI RefSeq microbial genome database) and aligned against ARG references using ResFinder (v4.1, based on the CARD database). Strong positive signals and negative controls from each batch were screened independently, with pathogen positivity criteria as follows: for bacteria (excluding mycobacteria), fungi, viruses, and parasites, positivity required either ≥3 species-level mapped reads with 50% primer coverage and absence in negative controls, or detected reads ≥10-fold higher than negative controls; for mycobacteria, positivity required either ≥1 detected read or reads ≥5-fold higher than negative controls. If the test results show the presence of two or more pathogens, it is classified as a mixed infection.

### 2.7 Statistical analysis

The median (interquartile range) [M(Q1, Q3)] was used to describe the distribution characteristics in cases of non-normal distribution. Group comparisons were performed using the χ² test. p-value < 0.05 was considered indicative of a statistically significant difference.

## 3 Results

### 3.1 Patient characteristics

This study enrolled a total of 166 patients suspected of pulmonary infection, who were recruited from March 2024 to April 2025. Specimens from all patients underwent both tNGS and TPDs. The baseline characteristics of the enrolled patients are summarized in **Table 1**.

**Table 1:** Clinical characteristics of the patients.

Characteristics	Patients (N = 166)
Gender (male/female)	88/78
Age, year, median [Q1-Q3]	52.00 [35.25-62.00]
Application of antibiotics before tNGS, n (%)	166 (100%)
White blood cells (10^9^), median [Q1-Q3]	6.26 [5.19-7.78]
Procalcitonin (ng/mL), median [Q1-Q3]	0.06 [0.03-0.10]
Interleukin (pg/mL), median [Q1-Q3]	5.42 [0.00-30.34]
C-reactive protein (mg/dl), median [Q1-Q3]	1.68 [0.00-28.99]

Q1, First Quartile; Q3, Third Quartile.

### 3.2 Pathogen detection using tNGS and TPDs

**Figure 2:**
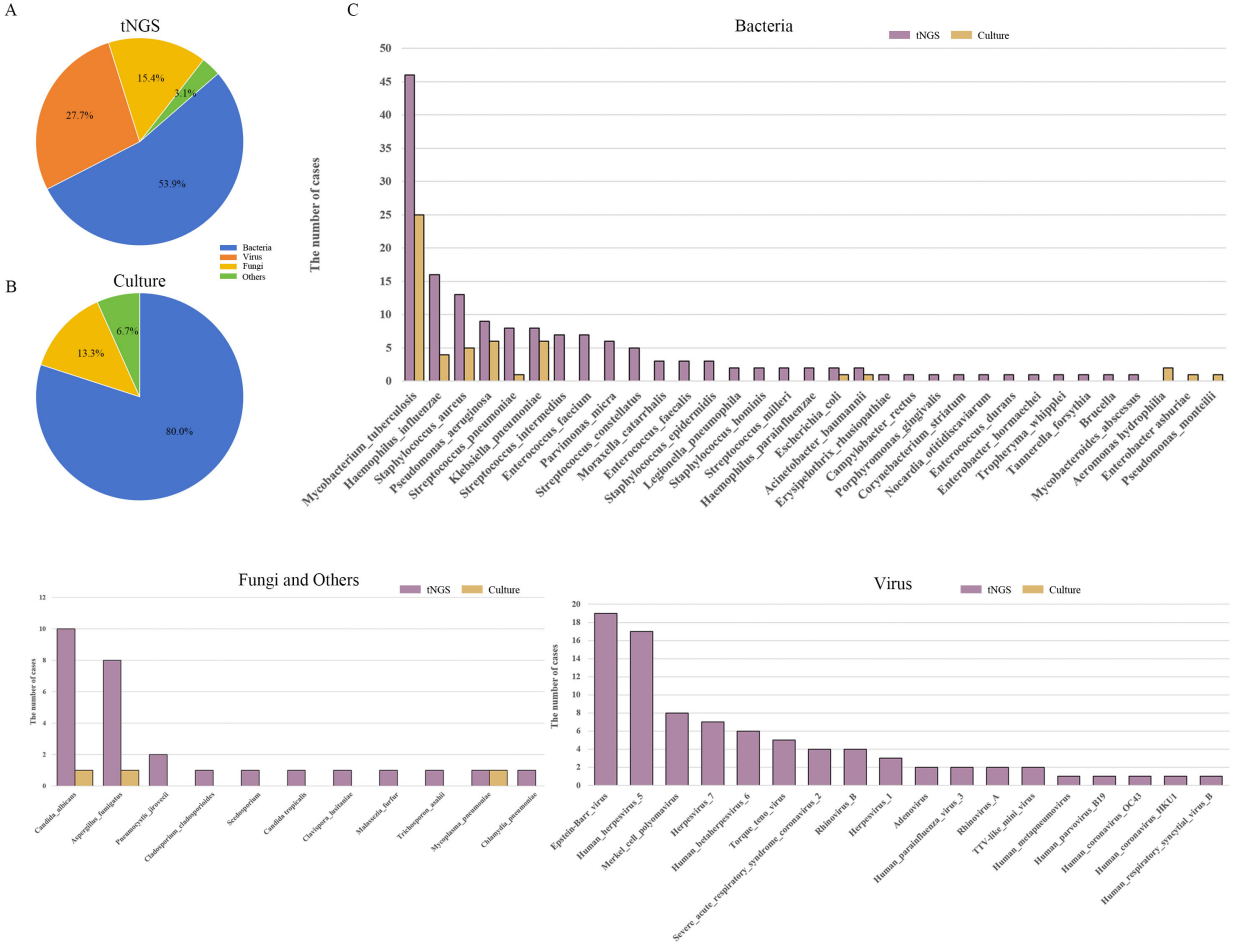
Analysis of pathogen distribution in pulmonary infections. **(A)** Types of pathogens identified by tNGS; **(B)** Types of pathogens identified by TPDs; **(C)** Types and frequency distribution of pathogens identified by tNGS and TPDs. tNGS, targeted next-generation sequencing; TPDs, traditional pathogen detection methods.

Among the 166 clinical samples submitted for testing, there were 89 fresh tissue, 52 BALF, 22 pleural effusions, and 3 sputum samples. tNGS testing identified a total of 65 pathogens across 135 cases, including 35 bacterial species (53.85%), 10 fungal species (15.38%), 18 viral species (27.69%), in addition to 1 case of *Mycoplasma pneumoniae* and a case of *Chlamydia pneumoniae* (**Figure 2A**). For bacterial detection, the three most prevalent pathogens were *Mycobacterium tuberculosis complex* (27.71%), *Haemophilus influenzae* (9.04%), and *Staphylococcus aureus* (7.23%). Among fungi, the most frequently detected species was *Candida albicans* (5.42%), followed by *Aspergillus fumigatus* (4.82%). For viruses, the top three identified pathogens were *Epstein-Barr virus* (11.45%), *Human herpesvirus 5* (10.24%), and *Merkel cell polyomavirus* (4.82%).

In contrast, the TPDs detected 14 pathogens across 54 cases, comprising 12 bacterial species (80.0%), 2 fungal species (13.3%), and 1 case of *Mycoplasma pneumoniae* (6.7%) (**Figure 2B**). Among the bacteria detected, the most prevalent was *Mycobacterium tuberculosis* (27.71%), followed by *Pseudomonas aeruginosa* and *Klebsiella pneumoniae* (both 3.61%). For fungi, only 1 case of *Candida albicans* and 1 case of *Aspergillus* (**Figure 2C**). Detailed information, including p sample types per patient and pathogens detection results, is provided in **Supplementary Table S2**.

### 3.3 Detection efficiency of tNGS and TPDs

**Figure 3:**
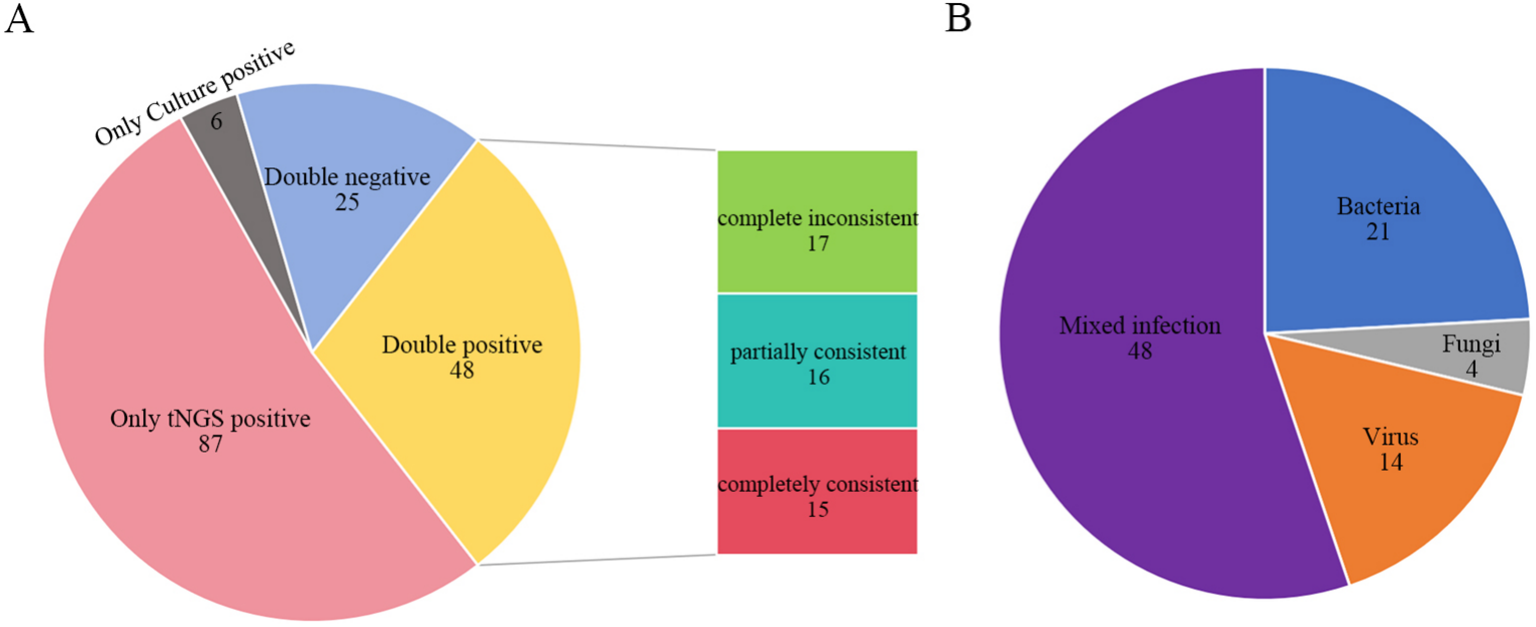
Comparison of tNGS and TPDs. **(A)** Consistency comparison of tNGS and TPDs result; **(B)** Infection types among patients positive only for tNGS. Complete inconsistent: TPDs and tNGS detected entirely different organisms from the same sample. Partially consistent: TPDs and tNGS detected some identical organisms while each also detected some unique organisms from the same sample. Completely consistent: TPDs and tNGS detected the same organisms from the same sample. tNGS, targeted next-generation sequencing; TPDs, traditional pathogen detection methods.

Among the 166 patients, TPDs identified pathogens in 54 cases, corresponding to an overall positive detection rate of 32.53% (54/166). In contrast, tNGS detected pathogens in 135 cases, with a notably higher overall positive detection rate of 81.33% (135/166). For tNGS, the positive detection rate for non-viral pathogens was 78.92% (131/166). The difference in the positive detection rate of non-viral pathogens between the two methods was statistically significant (χ² = 72.36, p < 0.001) (**Table 2**). Of all cases, 87 were positive exclusively by tNGS, while 6 were positive only through TPDs. Additionally, 25 cases were negative by both methods, and 48 cases tested positive through both. Among the 48 double positive cases, 17 showed complete inconsistency in pathogen identification, 16 showed partially consistency, and 15 showed complete consistency (**Figure 3A**). Within the group that tested positive only through tNGS, the rate of single pathogen detection was 44.83% (39/87), whereas the rate of mixed infections was 55.17% (48/87). Notably, the most common type of mixed infection was bacterial-viral coinfection, accounting for 31.25% (15/48) of these mixed infection cases (**Figure 3B**).

**Table 2:** Comparison of diagnostic performance between tNGS and TPDs for non-viral pathogens.

Method/Statistic	Positive, n (%)	Detection of pathogens, n (%)			Mixed infection
		Bacteria	Fungi	Others	
tNGS	131 (78.92)	106 (63.39)	25 (15.06)	2 (1.20)	39 (23.49)
TPDs	54 (32.53)	52 (31.33)	1 (0.60)	1 (0.60)	2 (1.20)
χ^2^	72.36	35.21	24.04	0.34	34.72
p-value	< 0.001	< 0.001	< 0.001	0.56	< 0.001

tNGS, targeted next-generation sequencing; TPDs, traditional pathogen detection methods.

### 3.4 Detection of ARGs by tNGS

tNGS identified a total of 16 ARGs. Specifically, 10 cases harbored the *ermB* gene, which confers resistance to macrolides, lincosamides (including lincosamycin), and streptogramins (MLS_8_ antibiotics). Additionally, 8 cases harbored the *TEM* gene, which mediates resistance to penicillin and cephalosporin, while 7 cases were carried the *tetQ* gene, responsible for resistance to tetracycline (**Table 3**).

**Table 3:** Detection of antibiotic resistance genes by tNGS.

Drug resistance type	ARGs	Number of patients
Penicillins and cephalosporins (which can be inhibited by β-lactamase inhibitors)	CTX-M-1	1
	OXA-2	1
	SHV	2
	TEM	8
Tetracycline	tetK	2
	tetM	3
	tetQ	7
	tetW	1
Macrolides, lincosamides and streptogramins	ermB	10
	mefA	4
	msrA	2
Aminoglycosides	AAC(6’)	1
	APH	4
Methicillin and all β-lactams that are structurally similar to methicillin	mecA	2
Chloramphenicol-class antibiotics	floR	1
Multidrug resistance via efflux	ADE	1

## 4 Discussion

The global incidence of infectious diseases is currently on the rise, with an increasing diversity and complexity of pathogens (Qian et al., 2020). Epidemiological surveys indicate that pulmonary infections rank among the most prevalent infectious diseases in clinical practice (Schlaberg et al., 2017). Accurate identification of the etiological agents of infection is crucial for the effective treatment and management of such diseases (Chen et al., 2020). Recent studies have demonstrated that tNGS exhibits high sensitivity in detecting respiratory pathogens, whereas TPDs often lack sufficient sensitivity and specificity to identify certain fungi and viruses (Guo et al., 2025). Notably, these specific fungi and viruses are increasingly recognized as significant contributors to pulmonary infections. Moreover, tNGS is capable of not only detecting bacteria but also identifying a broad spectrum of other pathogens, including fungi, viruses, parasites, *Mycoplasma* spp. and *Chlamydia* spp.

In this study, we employed a tNGS panel that targets 386 pathogenic microorganisms, encompassing bacteria, fungi, DNA/RNA viruses, and other pathogens, effectively covering those commonly associated with pulmonary infections. Unlike previous studies that primarily utilized BALF as the detection sample (Yin et al., 2024; Xu et al., 2025), this study incorporated multiple sample types, including fresh tissue, BALF, pleural effusion, and sputum. The pathogen detection rate of tNGS was 81.53%, which was significantly higher than the 32.53% detection rate by TPDs (p < 0.001), a result that further corroborates findings from previous research. Among pathogens detected by tNGS, the most prevalent ones were the *Mycobacterium tuberculosis complex* (27.71%), *Epstein-Barr virus* (11.45%), *Human herpesvirus 5* (10.24%), and *Haemophilus influenzae* (9.04%), indicating that bacteria remain the predominant pathogens in respiratory infections. In recent years, data from community-acquired pneumonia (CAP) cases within the Chinese population have shown a marked increase in viral pulmonary infections (Shang et al., 2020; Yin et al., 2023). In our study, viral infections were primarily observed as mixed infections with bacterial pathogens, which TPDs failed to detect. Collectively, these findings highlight tNGS as a valuable auxiliary tool for the routine detection of pulmonary infections, enhancing the sensitivity for identifying complex pathogens and providing novel insights for clinical disease management.

In this study, the patterns of mixed infections were notably diverse, comprising over half of the positive cases. This finding aligns well with previous reports (Ren et al., 2025; Tan et al., 2025). While controversies exist regarding whether mixed infections worsen clinical outcomes or prolong treatment duration, current evidence suggests that respiratory viral infections complicated by bacterial co-infections are frequently linked to severe clinical presentations, increased ICU admissions rates, and higher mortality (Liu et al., 2023; Santus et al., 2024). Therefore, the mixed infections observed in this study must be comprehensively evaluated, with integration of clinical symptoms, imaging results, and other relevant clinical data. Furthermore, tNGS identified 26 ARGs; however, it is important to note that the presence of these ARGs does not necessarily correlate with drug resistance phenotypes. For instance, a study of 41 patients with lower respiratory tract infections detected 183 ARGs through mNGS, but only 24 of these ARGs were consistent with the observed phenotypes, while 16 were completely inconsistent with phenotypic results (Charalampous et al., 2019). Notably, among the ARGs identified in this analysis, the *TEM* gene holds particular clinical significance as cephalosporins are frequently employed as “last-line therapeutic agents” in the treatment of infectious diseases, making them highly relevant in clinical settings. Therefore, the early identification of the *TEM* gene and other genes associated with cephalosporin resistance through tNGS can provide timely, actionable insights regarding potential drug resistance risks, guiding clinical decision-making. This proactive approach allows healthcare providers to adjust treatment strategies accordingly, such as avoiding the ineffective use of cephalosporins, thereby reducing the risk of treatment failure and limiting unnecessary antibiotic exposure. Collectively, ARGs data obtained from tNGS should be regarded as clinical references rather than definitive evidence of drug resistance. Validation of these ARGs findings through drug sensitivity testing is therefore essential, and such results must be integrated with clinical symptoms and other test data to ensure a comprehensive assessment of their clinical relevance.

Despite the valuable insights provided by this study, several limitations must be acknowledged. First, this research was a single-center prospective study within a narrow sample source range; therefore, large-scale multi-regional studies are necessary to further validate the generalizability of our findings. Second, although multiple types of samples were included in this study, these samples were not obtained from the same patients, which prevented effective comparison of the impact of different sample types on tNGS detection outcomes. Third, this study did not compare tNGS with quantitative real-time polymerase chain reaction (qPCR)—the most commonly used clinical molecular detection method. This gap means we could not further clarify the advantages and differences of tNGS over routine clinical molecular detection in terms of detection performance, nor could we evaluate its feasibility for replacing or supplementing qPCR in clinical practice. Additionally, due to the critical condition of some patients, empirical antibiotic treatment was administered before sample collection. This factor may have inhibited the growth of bacteria and fungi; thereby affecting the positive rate of TPDs results; however, it had no significant interference with tNGS-based nucleic acid detection. This may have, to a certain extent, exaggerated the difference in detection sensitivity between tNGS and TPDs, making it impossible to completely rule out the impact of this factor on the performance comparison between the two methods.

## 5 Conclusion

As an emerging diagnostic technology, tNGS has demonstrated significant promise in identifying pathogens responsible for pulmonary infections. Compared with TPDs, tNGS offers enhanced sensitivity, a broader pathogen detection spectrum, and superior ability to detect mixed infections. Furthermore, it enables the detection of ARGs, solidifying its roles as a crucial tool for accurate clinical diagnosis. This technological advancement holds the potential to improve patient outcomes by facilitating more personalized and effective treatment strategies.

## Data Availability

The original contributions presented in the study are included in the article/**Supplementary Material**. Further inquiries can be directed to the corresponding author.

## References

[B1] Almas S. Carpenter R. E. Singh A. Rowan C. Tamrakar V. K. Sharma R. (2023). Deciphering microbiota of acute upper respiratory infections: A comparative analysis of PCR and mNGS methods for lower respiratory trafficking potential. Adv. Respir. Med. 91, 49–65. doi: 10.3390/arm91010006, PMID: 36825940 PMC9952210

[B2] Charalampous T. Kay G. L. Richardson H. Aydin A. Baldan R. Jeanes C.. (2019). Nanopore metagenomics enables rapid clinical diagnosis of bacterial lower respiratory infection. Nat. Biotechnol. 37, 783–792. doi: 10.1038/s41587-019-0156-5, PMID: 31235920

[B3] Chen L. Liu W. Zhang Q. Xu K. Ye G. Wu W.. (2020). RNA based mNGS approach identifies a novel human coronavirus from two individual pneumonia cases in 2019 Wuhan outbreak. Emerg. Microbes Infect. 9, 313–319. doi: 10.1080/22221751.2020.1725399, PMID: 32020836 PMC7033720

[B4] Chen Y. Fan L. C. Chai Y. H. Xu J. F. (2022). Advantages and challenges of metagenomic sequencing for the diagnosis of pulmonary infectious diseases. Clin. Respir. J. 16, 646–656. doi: 10.1111/crj.13538, PMID: 36068680 PMC9527156

[B5] Febbo J. Dako F. (2024). Pulmonary infection. Clin. Chest Med. 45, 373–382. doi: 10.1016/j.ccm.2024.02.009, PMID: 38816094

[B6] Gu W. Deng X. Lee M. Sucu Y. D. Arevalo S. Stryke D.. (2021). Rapid pathogen detection by metagenomic next-generation sequencing of infected body fluids. Nat. Med. 27, 115–124. doi: 10.1038/s41591-020-1105-z, PMID: 33169017 PMC9020267

[B7] Guo X. Xie N. Xi X. Li P. Jia J. Chen L.. (2025). Clinical application of targeted next-generation sequencing utilizing bronchoalveolar lavage fluid in thoracic surgery ICU patients with suspected pulmonary infections. J. Appl. Microbiol. 136, lxae313. doi: 10.1093/jambio/lxae313, PMID: 39741395

[B8] Liu Y. N. Zhang Y. F. Xu Q. Qiu Y. Lu Q. B. Wang T.. (2023). Infection and co-infection patterns of community-acquired pneumonia in patients of different ages in China from 2009 to 2020: a national surveillance study. Lancet Microbe 4, e330–e339. doi: 10.1016/s2666-5247(23)00031-9, PMID: 37001538 PMC12514336

[B9] Qian Y. Y. Wang H. Y. Zhou Y. Zhang H. C. Zhu Y. M. Zhou X.. (2020). Improving pulmonary infection diagnosis with metagenomic next generation sequencing. Front. Cell Infect. Microbiol. 10. doi: 10.3389/fcimb.2020.567615, PMID: 33585263 PMC7874146

[B10] Ren G. Ma L. Yan C. Xue Q. Zhang H. Wang W.. (2025). Application of targeted metagenomic next-generation sequencing in pneumonia patients. Microbiol. Spectr. 13, e01713–e01724. doi: 10.1128/spectrum.01713-24, PMID: 40548737 PMC12323347

[B11] Santus P. Danzo F. Signorello J. C. Rizzo A. Gori A. Antinori S.. (2024). Burden and risk factors for coinfections in patients with a viral respiratory tract infection. Pathogens. 13, 993. doi: 10.3390/pathogens13110993, PMID: 39599546 PMC11597400

[B12] Schlaberg R. Chiu C. Y. Miller S. Procop G. W. Weinstock G. (2017). Validation of metagenomic next-generation sequencing tests for universal pathogen detection. Arch. Pathol. Lab. Med. 141, 776–786. doi: 10.5858/arpa.2016-0539-RA, PMID: 28169558

[B13] Shang L. Xu J. Cao B. (2020). Viral pneumonia in China: from surveillance to response. Lancet Public Health 5, e633–e634. doi: 10.1016/s2468-2667(20)30264-4, PMID: 33271074 PMC7834624

[B14] Tan J. Chen Y. Lu J. Lu J. Liu G. Mo L.. (2025). Pathogen distribution and infection patterns in pediatric severe pneumonia: a targeted next-generation sequencing study. Clinica Chimica Acta 565, 119985. doi: 10.1016/j.cca.2024.119985, PMID: 39362455

[B15] Xu Q. Chen Q. Qiu W. Liu L. Zeng W. Chen J.. (2025). Application of targeted next-generation sequencing for pathogens diagnosis and drug resistance prediction in bronchoalveolar lavage fluid of pulmonary infections. Front. Cell Infect. Microbiol. 15. doi: 10.3389/fcimb.2025.1590881, PMID: 40552119 PMC12183283

[B16] Yin L. Zhang Y. Zheng Y. Luo Q. Zhao L. Ni W.. (2023). Early detection of aspergillus species in lower respiratory tract is associated with higher mortality in viral community-acquired pneumonia: A multicenter prospective cohort study in China. Lung. 201, 387–396. doi: 10.1007/s00408-023-00638-2, PMID: 37480410

[B17] Yin Y. Zhu P. Guo Y. Li Y. Chen H. Liu J.. (2024). Enhancing lower respiratory tract infection diagnosis: implementation and clinical assessment of multiplex PCR-based and hybrid capture-based targeted next-generation sequencing. EBioMedicine. 107, 105307. doi: 10.1016/j.ebiom.2024.105307, PMID: 39226681 PMC11403251

